# Integrating biological pathway polygenic scores and trauma in psychosis: findings from the EU-GEI study

**DOI:** 10.1038/s41398-026-04317-7

**Published:** 2026-08-05

**Authors:** Giulia Trotta, Isabelle Austin-Zimmerman, Edoardo Spinazzola, Lucia Sideli, Monica Aas, Victoria Rodriguez, Senta Haussler, Zhikun Li, Perry BM Leung, Qiang Li, Diego Quattrone, Teya Petrova, Craig Morgan, Michael O’Donovan, Pak C. Sham, Evangelos Vassos, Chloe CY Wong, Richard Bentall, Robin M. Murray, Luis Alameda, Marta Di Forti

**Affiliations:** 1 Social, Genetic and Developmental Psychiatry Centre, Institute of Psychiatry, Psychology and Neuroscience, King’s College London, London, UK; 2 South London and Maudsley NHS Foundation Trust, London, UK; 3 NIHR Biomedical Research Centre, London, UK; 4 Department of Psychosis Studies, Institute of Psychiatry, Psychology and Neuroscience. King’s College London, London, UK; 5 Department of Human Science, LUMSA University, Rome, Italy; 6 Camden Early Intervention Service, North London NHS Foundation Trust, London, UK; 7 Department of Psychiatry, School of Clinical Medicine, LKS Faculty of Medicine, The University of Hong Kong, Hong Kong SAR, China; 8 Service of General Psychiatry, Treatment and Early Intervention in Psychosis Program, Lausanne. University Hospital (CHUV), Lausanne, Switzerland; 9 ESRC Centre for Society and Mental Health, Institute of Psychiatry, King’s College London, London, UK; 10 Centre for Neuropsychiatric Genetics and Genomics, Division of Psychological Medicine and Clinical Neurosciences, Cardiff University, Cardiff, UK; 11 Department of Psychology, University of Sheffield, Sheffield, UK; 12 Centro Investigacion Biomedica en Red de Salud Mental (CIBERSAM); Instituto de Biomedicina de Sevilla (IBIS), Hospital Universitario Virgen del Rocio, Departamento de Psiquiatria, Universidad de Sevilla, Sevilla, Spain

**Keywords:** Schizophrenia, Clinical genetics

## Abstract

Psychotic disorders are complex, multifactorial conditions influenced by both genetic liability and early environmental adversity. Polygenic risk scores (PRSs) derived from genome-wide association studies have shown utility in capturing genetic predisposition, but their biological interpretability remains limited. In this study, we evaluated whether biologically informed pathway-specific polygenic scores (pPGSs) for psychosis, restricted to neurotransmitter-related pathways, could help clarify gene-environment interplay. Using data from 1 192 individuals in the EU-GEI multi-site case-control study, we constructed pPGSs for dopamine, glutamate, GABA, and serotonin systems. We investigated associations between pPGSs and childhood trauma (rGE), their interactions on psychosis risk (GxE), and the influence of the genome-wide psychosis PRS on these relationships. Serotonin, dopamine, and glutamate pPGSs were positively associated with a composite trauma exposure (i.e., abuse and neglect), suggesting shared genetic factors contributing to both psychosis liability and early adversity. Significant negative GxE effects were observed for both dopamine and serotonin pPGSs, indicating that higher trauma exposure diminished the relative influence of genetic liability on psychosis risk. Adjustment for the genome-wide psychosis PRS attenuated most effects, but serotonergic and dopaminergic associations remained robust, supporting pathway-specific contributions beyond general polygenic risk. These findings provide proof-of-concept for the utility of pPGSs in psychiatric research, suggesting both genetic contributions to trauma exposure and GxE effects on psychosis risk. Further research incorporating epigenetic data and longitudinal designs may enhance mechanistic insight and translational potential.

## Introduction

Psychotic disorders, including schizophrenia and bipolar disorder, are severe mental health conditions with complex aetiologies and substantial heritability [1]. Polygenic risk scores (PRSs) have become key tools in quantifying genetic liability for psychiatric conditions, using data from genome-wide association studies (GWAS) to summarise total genetic risk based on common variants across the genome [2]. While genome-wide PRSs have shown robust associations with psychosis and related traits, their interpretability is limited, as they aggregate across biologically heterogeneous loci [3].

Recent advances have led to the development of pathway-specific polygenic scores (pPGSs), which restrict genetic risk to genes within defined biological systems [4]. By mapping GWAS signals onto functional gene sets, pPGSs offer a biologically grounded approach to clarifying the genetic architecture of complex disorders [5]. This strategy enables investigation of risk distributed across molecular pathways and supports testing of more targeted mechanistic hypotheses. Although pPGSs may enhance interpretability, it remains unclear to what extent they provide additional insight beyond traditional genome-wide PRSs and established environmental risk factors [6].

In the context of psychosis, four neurotransmitter systems have consistently been implicated in both aetiopathogenesis and treatment response: dopamine, glutamate, gamma-aminobutyric acid (GABA), and serotonin [7]. Dopaminergic dysregulation remains a core hypothesis to account for positive symptoms and antipsychotic efficacy, while glutamatergic dysfunction, especially involving N-methyl-D-aspartate (NMDA) receptor signalling, has been linked to cognitive deficits and negative symptoms [8–10]. GABAergic signalling underlies cortical inhibitory control and has been associated with sensory gating and disorganisation [11, 12]. Serotonin modulates both dopaminergic and glutamatergic systems and contributes to mood and possibly indirectly to perceptual disturbances [13, 14]. Each system is a pharmacological target of existing or emerging treatments for psychotic disorders, further supporting their relevance to clinical outcomes [15].

Accumulating evidence indicates that childhood trauma, in the form of abuse and neglect, is a robust environmental risk factor for both affective and non-affective forms of psychosis, including schizophrenia and bipolar disorder [16–19]. It has been estimated that preventing childhood trauma could reduce the overall incidence of psychosis by up to 30% [20]. Meta-analytic and family-based studies suggest a gene-environment correlation (rGE) between childhood trauma and schizophrenia PRS [21], while Mendelian randomisation analyses indicate a causal role of childhood trauma in psychosis, depression, and attention deficit hyperactivity disorder (ADHD) after accounting for rGE [22].

Building on this evidence, the current study examined childhood trauma in relation to neurotransmitter-related pPGSs for dopamine, glutamate, GABA, and serotonin, derived from a cross-disorder GWAS meta-analysis of schizophrenia and bipolar I [23, 24]. We investigated: (1) associations between pPGSs and childhood trauma (rGE); (2) interactions between pPGSs and trauma exposure on psychosis risk (GxE); and (3) the extent to which these pathway specific effects were independent of genome-wide psychosis polygenic liability. Given the limited literature on pPGSs, our hypotheses were necessarily speculative. We expected that dopaminergic and serotonergic pPGSs would show the strongest associations with trauma exposure and significant GxE effects on psychosis risk, consistent with their central role in stress responsivity and neurotransmission [25].

## Materials and methods

### Study design and participants

This study utilised data from Work Package 2 (WP2) of the European Network of National Schizophrenia Networks Studying Gene-Environment Interactions (EU-GEI) project. This was a multi-centre case-control study examining genetic and environmental risk factors for psychosis [26–29]. The sample consists of first episode psychosis (FEP) cases and unaffected controls, recruited across 16 sites in Europe and Brazil. Participants were aged 18–64 years and were included if they presented with a first episode of a psychotic disorder, confirmed using the Operational Criteria Checklist for Psychotic and Affective illness (OPCRIT) [28, 30]. Exclusion criteria were prior treatment for psychosis, organic psychotic disorders (ICD-10: F09), and transient psychotic symptoms resulting from acute intoxication (ICD-10: F1X.5). Control participants, recruited from the same catchment areas, were screened to exclude individuals with a lifetime psychotic disorder [27]. All participants provided written informed consent, and the study received approval from relevant ethical committees.

### Measures of childhood trauma

Childhood trauma was assessed using the 28-item short-form version of the Childhood Trauma Questionnaire (CTQ-SF), a retrospective self-report measure evaluating exposure to physical, emotional, and sexual abuse, and emotional and physical neglect [31]. Responses were rated on a 5-point Likert scale from *Never True (1)* to *Very Often True (5)*, yielding a total CTQ score as well as domain-specific sub-scores (i.e., emotional abuse, physical abuse, sexual abuse, emotional neglect, and physical neglect). Following the same methodological approach as previous studies examining the impact of childhood trauma on psychosis and related outcomes [32–34], exposure was defined as 0 = ‘absent’ or 1 = ‘present’ based on moderate to severe cut-off scores: ≥ 13 for emotional abuse, ≥ 10 for physical abuse, ≥ 8 for sexual abuse, ≥ 15 for emotional neglect and ≥ 10 for physical neglect. A Composite Score (range 0–5) was then derived by summing binary indicators for all five CTQ domains (three abuse and two neglect subscales), providing an index of cumulative maltreatment. This served as the primary trauma variable. In sensitivity analyses, we also derived: (1) an Abuse-Only Score (range 0–3) summing only the three abuse domains, and (2) a Neglect-Only Score (range 0–2) summing the two neglect domains. These narrower indices allowed us to test whether observed effects were driven predominantly by abuse-related or neglect-related experiences. Finally, all main analyses were repeated using the raw continuous CTQ total, abuse-only, and neglect-only scores to evaluate robustness of findings across trauma operationalisations and to allow comparability.

### Measures of outcome

The primary outcome was defined as case status, meaning a diagnosis of psychosis defined as ICD-10 codes F20-29 or psychotic forms of affective disorders within F30-33 (manic, bipolar, or depressive episodes with psychotic features). Research-based diagnoses were primarily established using the Operational Criteria Checklist (OPCRIT) [28, 30]. The OPCRIT system is a standardised diagnostic tool that enables structured assessment of psychiatric symptoms and facilitates algorithm-based diagnostic classification across multiple systems (e.g., ICD-10, DSM-IV). It comprises a 96-item checklist that can be completed based on a semi-structured clinical interview and/or through review of clinical notes and collateral sources. OPCRIT assessments allowed for the evaluation of pre-morbid functioning, symptomatology, and longitudinal course. The reliability of OPCRIT-based diagnoses across study sites was acceptable, with an inter-rater agreement of κ = 0.7, indicating good diagnostic consistency across different clinical settings. In cases where OPCRIT data were incomplete or unavailable, clinical diagnoses provided by treating psychiatrists were used as a secondary source of outcome classification. This ensured maximisation of available data while maintaining diagnostic validity across the sample.

### Genotyping and polygenic score construction

Genotyping was conducted at the MRC Centre for Neuropsychiatric Genetics and Genomics, Cardiff University (UK) using the Illumina HumanCoreExome-24 BeadChip. Standard EU-GEI quality control procedures included: (1) exclusions of SNPs with call rates < 98%, minor allele frequency < 1%, or Hardy-Weinberg equilibrium p < 1×10^−6^ in controls; (2) population stratification control, using principal component analysis (PCA); (3) exclusion of individuals with evidence of relatedness, to ensure independence of observations (KING *r*^2^ > 0.044). We calculated genetic principal components (PCs) using 1000 Genomes data as the reference populations [35]. Analyses were restricted to individuals of confirmed European ancestry, for whom polygenic scores were generated using PRS-CS, a Bayesian regression framework that accounts for linkage disequilibrium patterns and shrinkage priors to improve effect-size estimation [36].

As first step, a genome-wide PRS indexing overall psychosis liability was created using summary statistics from a cross-disorder fixed-effects meta-analysis combining the most recent GWAS of schizophrenia and bipolar I [23, 24]. The meta-analysis was conducted using METAL, retaining variants present in either input GWAS [37]. LD Score Regression (LDSC) was used to estimate genomic inflation and confirm no significant sample overlap [38]. Only bipolar I was included because psychotic features occur in approximately 55-174% cases [39–41], and prior evidence indicates that the schizophrenia PRS predicts bipolar I but not bipolar II [42]. The EU-GEI target sample was excluded from discovery GWAS to ensure dataset independence. The resulting PRS was standardised (mean = 0, SD = 1) and used as an additional covariate and reference score in downstream analyses [43].

Secondly, four pPGSs were derived to capture genetic liability related to major neurotransmitter systems implicated in psychosis: dopamine, glutamate, GABA, and serotonin. These pathways were selected based on longstanding evidence of their relevance to psychiatric disorders and treatment mechanisms [6]. These pPGSs represent pathway-restricted versions of the psychosis PRS derived using PRS-CS. Specifically, SNP effect sizes estimated by PRS-CS from the schizophrenia – bipolar I meta-analysis were used as the base weights, and scoring was restricted to SNPs mapping to genes within each neurotransmitter pathway. Genes for each pathway were identified using three established biological databases: KEGG, REACTOME, and AmiGO [44–46]. These databases provide curated gene sets relevant to specific biological functions. Complete gene list is available in the Supplement [6]. For each pathway, all single nucleotide polymorphisms (SNPs) located within the genomic boundaries of pathway genes were extracted from Ensembl (release 111, GRCh37). Only SNPs with valid reference SNP identifiers (rsIDs) were retained. Redundant entries were removed, and the resulting SNP lists were formatted for compatibility with downstream scoring software. The final SNP lists for each neurotransmitter pathway were used to generate pPGSs via PRSet, a module within PRSice-2 that enables pathway-based polygenic scoring [5]. The same cross-disorder schizophrenia-bipolar I served as the discovery dataset for all four neurotransmitter pathways, ensuring methodological consistency. No additional LD clumping or p-value thresholding was applied, as scoring relied on predefined SNP sets. All pPGSs were standardised (mean = 0, SD = 1) before analysis [43].

### Statistical analyses

All analyses were conducted in R (version 4.3.1). We first visualised the distribution of pPGSs (standardised to z-scores) and examined pairwise correlations using Pearson’s r. This step helped assess collinearity and determine shared versus distinct variance components across scores. To evaluate associations between pPGSs and childhood trauma, we used linear regression models with three trauma outcomes: (1) a Composite Trauma Score including abuse (i.e., emotional, physical and sexual) and neglect (i.e., emotional and physical); (2) an Abuse-Only Score; and (3) a Neglect-Only Score. All regression models were adjusted for sex, age, study site, and the first 10 ancestry principal components.

We computed both raw and adjusted R^2^ values, reporting variance explained across pPGSs. To account for potential bias due to differences in pathway size (i.e., the number of genes included in each pPGS), we performed an additional adjustment by regressing raw R^2^ values on pathway genes count within each trauma outcome and extracting the residuals [47]. These residualised R^2^ values (size-adjusted R^2^) reflect variance explained beyond that expected by pathway size. Scores were stratified into quintiles based on their empirical distribution in the full analytic sample. Quintiles were included as a categorical predictor in a single logistic regression model per pathway, adjusted for covariates, and marginal predicted probabilities were estimated from these models. Estimated probabilities of psychosis were plotted across pPGS quintiles to visualise dose-response trends. These values represent estimated probabilities based on the fitted model and are not predictive performance metrics. To test for GxE, we fitted logistic regression models predicting psychosis case-control status as the dependent variable, including main effects for pPGSs and trauma scores, as well as their interaction terms. The three trauma scores were tested separately. We evaluated the interaction terms and visualised interaction effects via predicted probability plots stratified by trauma level. Predicted probability of psychosis were obtained from fitted logistic regression models by converting model-based log-odds into probabilities. Model performance was assessed using Nagelkerke R^2^ (ΔR^2^) and likelihood ratio tests. To account for differences in pathway size, ΔR^2^ values were residualised on the number of genes per pathway. Hierarchical models were constructed beginning with a covariate-only model (M0), followed by the sequential addition of trauma exposure (M1), psychosis PRS (M2), and pPGSs (M3). As sensitivity analysis, all primary models were repeated using raw continuous CTQ scores (i.e., total, abuse-only, and neglect-only) instead of the cumulative indices to test robustness of findings across trauma operationalisations. In additional analyses, models were repeated with the genome-wide psychosis PRS included as an additional covariate to assess whether pathway specific effects were independent of overall polygenic liability. To further assess the potential impact of rGE, additional sensitivity analyses were conducted in which childhood trauma scores were residualised on the corresponding pPGS and covariates prior to testing GxE [48].

False Discovery Rate (FDR) correction using the Benjamini-Hochberg procedure was applied across all regression and interaction models to control for multiple comparisons [49].

## Results

### Sample characteristics

The primary analytic sample comprised 1 192 individuals of European ancestry, selected based on genetic principal component analysis (see Table 1). Cases (N = 438) and controls (N = 754) differed across multiple sociodemographic and trauma-related variables, in ways expected based on previous research. Cases were more likely to be men (63%) compared with controls (46%). The mean age was also lower among cases (M = 31, SD = 11) relative to controls (M = 37, SD = 14). Country of recruitment was unequally distributed across groups, with a greater proportion of controls recruited in Brazil and the UK, while a higher proportion of cases were recruited in the Netherlands. Across all trauma types, exposure was more frequent among cases than controls. The largest differences were observed for emotional neglect (25% in cases vs. 12% in controls), emotional abuse (20% vs. 9%), and physical neglect (25 vs. 10%).

**Table 1:** Demographic Characteristics of the EU-GEI Sample.

Characteristic	Total sample N = 1 192	Controls N = 754	Cases N = 438	*p*-value
Gender				< 0.001
*Female*	568 (48%)	405 (54%)	163 (37%)	
*Male*	624 (52%)	349 (46%)	275 (63%)	
Age	35 (13)	37 (14)	31 (11)	< 0.001
Country				< 0.001
*Brazil*	299 (25%)	205 (27%)	94 (21%)	
*France*	122 (10%)	92 (12%)	30 (6.8%)	
*Holland*	262 (22%)	138 (18%)	124 (28%)	
*Italy*	135 (11%)	79 (10%)	56 (13%)	
*Spain*	102 (8.6%)	49 (6.5%)	53 (12%)	
*UK*	272 (23%)	191 (25%)	81 (18%)	
Emotional Abuse	152 (13%)	65 (8.6%)	87 (20%)	< 0.001
Physical Abuse	101 (8.5%)	41 (5.4%)	60 (14%)	< 0.001
Sexual Abuse	94 (7.9%)	38 (5.0%)	56 (13%)	< 0.001
Emotional Neglect	204 (17%)	93 (12%)	111 (25%)	< 0.001
Physical Neglect	184 (15%)	76 (10%)	108 (25%)	< 0.001

*P*-values from Wilcoxon rank-sum tests (continuous variables) and χ² tests (categorical variables; Fisher’s exact where appropriate).

### Pathway polygenic scores and trauma exposure (Gene-environment correlation)

To assess the association between pPGSs and trauma exposure, we conducted linear regression models adjusted for sex, age, study site, and ten ancestry PCs.

**Fig. 1:**
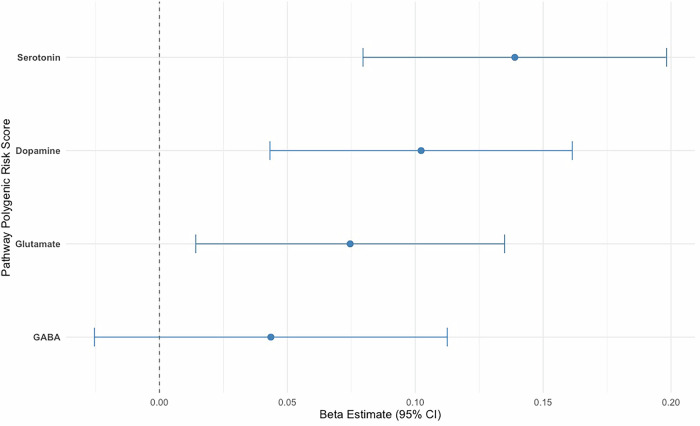
Forest Plot of pPGSs – Trauma Associations. Beta Estimate and 95% confidence intervals (CIs) from linear regression models examining associations between pPGSs and trauma exposure (Composite Score). All models are adjusted for age, gender, study site, and the first 10 genetic principal components. Points represent beta coefficients, with horizontal bars indicating the 95% CI.

For the Composite Score, three of the four pPGSs were positively associated with exposure to trauma, with standardised β coefficients ranging from 0.044 (GABA; SE = 0.035, p = 0.216, FDR = 0.216)–0.139 (serotonin; SE = 0.030, p < 0.001, FDR = < 0.001) (Fig. 1). Significant associations were observed for serotonin, dopamine, and glutamate pPGSs, while the GABA pPGS was not significant. Results for the Abuse-Only and Neglect-Only scores, which showed similar patterns, together with full regression results, are reported in the Supplementary Material (Figures S3A and S3B). The variance explained by each pPGS is presented in the Supplementary Material (Figure S4), showing that serotonergic and glutamatergic pPGSs account for the highest proportion of variance.

### Pathway polygenic scores quintile effects on psychosis risk

**Fig. 2:**
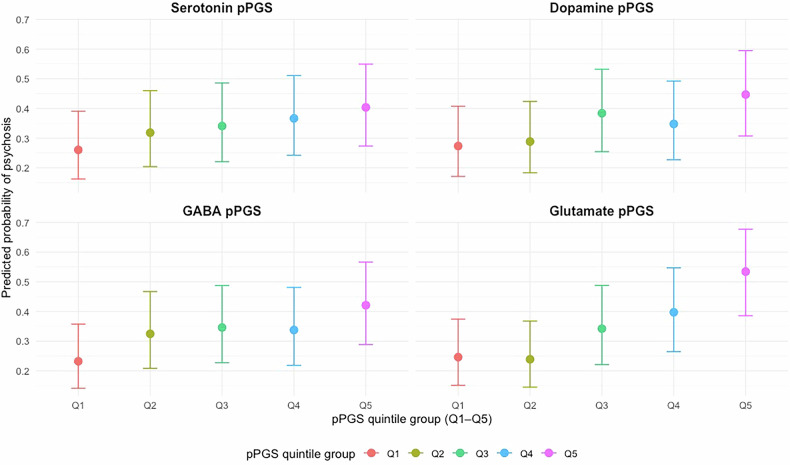
Quintile Effects of Biological Pathway Polygenic Risk Scores on Predicted Probability of Psychosis. Predicted probabilities of psychosis by quintile of each pPGS (serotonin, dopamine, glutamate, GABA), estimated using logistic regression models adjusted for age, sex, site, and ten ancestry principal components, rather than observed proportions. Quintiles were defined based on the distribution of each pPGS in the full analytic sample. Points represent predicted probabilities with 95% confidence intervals. Quintile plots are intended to illustrate distributional trends rather than formal statistical comparisons between groups.

To visualise the distributional impact of pPGSs on psychosis risk, we estimated predicted probabilities of psychosis across pPGS quintiles for each biological pathway (Fig. 2). For all four pPGSs – serotonin, dopamine, glutamate, and GABA – the predicted probabilities of psychosis were increased in the highest quintile (Q5) vs. the lowest quintile (Q1). However, confidence intervals overlapped substantially across intermediate quintiles for all pathways, indicating modest effect sizes and limited separation between groups. A clear dose-response pattern was evident for the glutamate pPGS, with predicted probabilities increasing from 0.25 (95% CI: 0.15 – 0.38) in Q1 to 0.52 (95% CI: 0.37 – 0.67) in Q5. The dopamine pPGS was also associated with an upward trend, rising from 0.28 (95% CI: 0.17 – 0.41) in Q1 to 0.43 (95% CI: 0.29 – 0.58) in Q5. A similar, though slightly attenuated, trend was observed for the serotonin pPGS, from 0.25 (95% CI: 0.16 – 0.39) to 0.40 (95% CI: 0.26 – 0.54). By contrast, the GABA pPGS showed a flatter profile, with less pronounced differences in predicted risk across quintiles, and overlapping confidence intervals across quintiles.

### Pathway polygenic scores x trauma interactions on psychosis risk

**Fig. 3:**
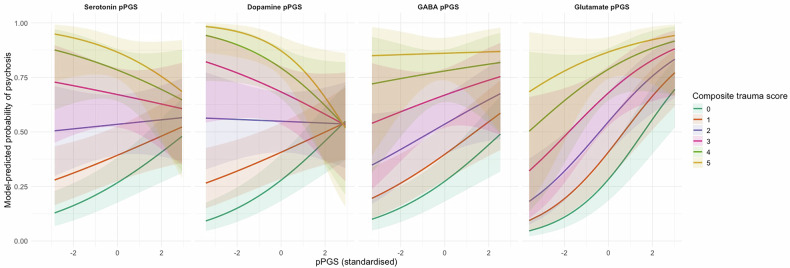
pPGSs × Composite Trauma Score Interaction on Psychosis Risk. Predicted probability of psychosis across the full distribution of standardised pPGS, stratified by level of trauma exposure. Each line represents a different trauma level (0-15 adversities). Confidence intervals are shaded. Across all pathways, higher trauma exposure is associated with greater psychosis risk (from pink to red lines). For the dopamine and serotonin pathways, individuals with high trauma exposure show a flatter slope across pPGS values, suggesting a negative GxE (i.e., the effect of genetic liability diminishes at higher trauma levels).

**Fig. 4:**
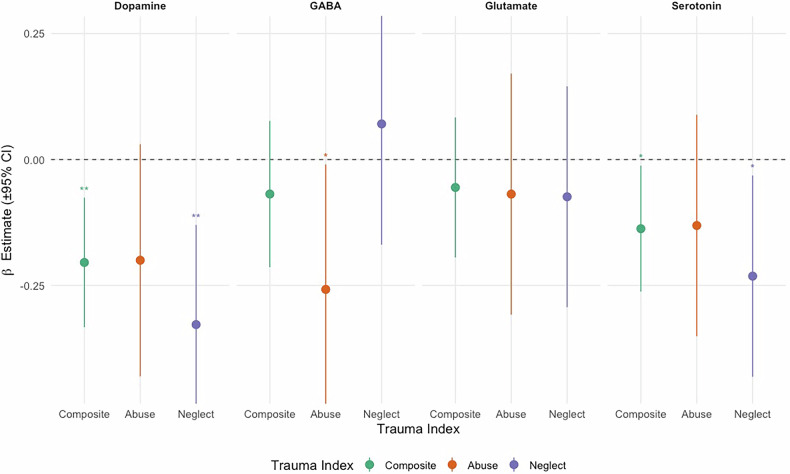
*Comparison of GxE Effects Across Trauma Indices.* Forest plot comparing beta estimates (±95% CI) for the interaction between pPGSs and different childhood trauma indices (Composite, Abuse-Only, and neglect-Only) on psychosis risk. Each panel corresponds to one neurotransmitter pathway (dopamine, GABA, glutamate, serotonin). The plot illustrates the relative strength and direction of GxE effects across trauma types, showing largely consistent negative associations except for a trend-level difference in the GABA pathway (*p* = 0.06).

We next tested whether pPGSs moderated the association between trauma exposure and psychosis risk. For the Composite Score, there was some evidence of interactions for both the dopamine (β = −0.204, SE = 0.0657, p = 0.002, FDR = 0.003) and serotonin (β = −0.137, SE = 0.0638, p = 0.031, FDR = 0.0042) pPGSs. These negative coefficients indicate that the association between polygenic liability and psychosis was attenuated at higher levels of trauma exposure (Fig. 3). When examining trauma dimensions separately, a nominal negative interaction between GABA pPGS and Abuse-Only Score emerged (β = −0.258, SE = 0.127, p = 0.04, FDR = 0.067), whereas no GABA interaction was detected for Neglect-Only exposure. The full results (Table S2) together with sensitivity analyses for the Abuse-Only and Neglect-Only scores (Figures S6A and S6B) are reported in the Supplementary Materials. Direct comparison across trauma indices revealed no statistically significant differences in GxE effect size between Abuse-Only and Neglect-Only models (all p > 0.05), although a trend-level difference was observed for the GABA pathway (p = 0.06) (Fig. 4).

Model performance analyses using Nagelkerke R^2^ and incremental (ΔR^2^) estimates, detailed in Supplementary Table S3 and Figure S7, confirmed that the inclusion of pPGSs modestly improved model fit, with the largest incremental variance observed for the glutamatergic and serotonergic pathways.

### Psychosis PRS and adjusted pathway analyses

**Fig. 5:**
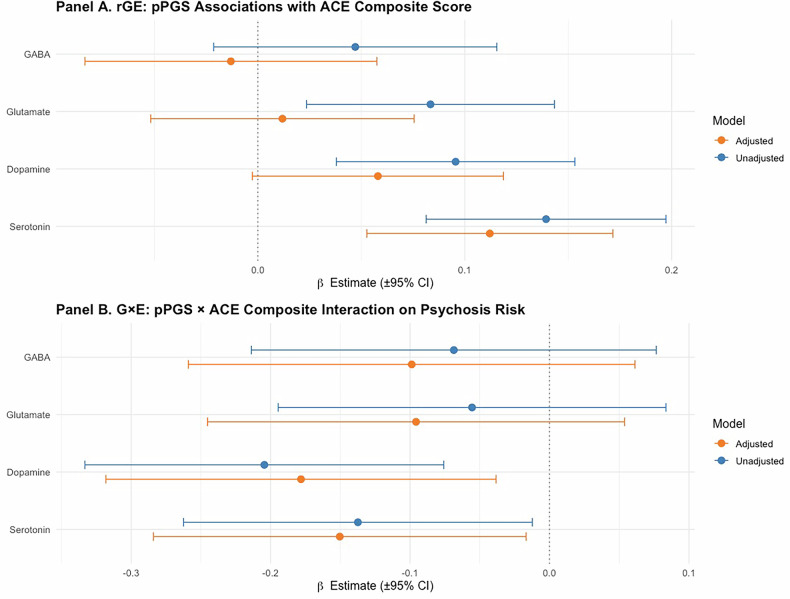
Comparison of Unadjusted and Psychosis PRS-Adjusted Analyses for the Composite Score. Points indicate beta estimates from rGE (Panel **A**) and GxE (Panel **B**) models, with horizontal bars representing 95% CIs. Blue points and CIs represent estimates from models adjusted for age, sex, study site, and the first 10 genetic PC (unadjusted for psychosis PRS). Orange dots and CIs represent models that additionally included psychosis PRS as covariate.

Analyses using the Psychosis PRS showed significant positive associations with Composite Score (β = 0.331, SE = 0.055, p < 0.001). In contrast, no significant psychosis PRS x Trauma interactions on psychosis risk were observed after FDR correction. Analyses including the other Trauma indices are reported in the Supplementary Materials. To determine whether pPGS associations were independent of genome-wide psychosis liability, all rGE and GxE models were re-run including the psychosis PRS as an additional covariate. The results, summarised in Fig. 5 and detailed in Supplementary Section 4, showed that adjustment for the psychosis PRS attenuated the magnitude of most effects. However, serotonergic and dopaminergic pPGSs remained significantly associated with Composite Score and continued to show negative interaction effects on psychosis risk.

## Discussion

This study is the first to explore whether pPGSs, derived from a cross-disorder base-GWAS capturing shared schizophrenia-bipolar I genetic liability and targeting neurotransmitter-specific gene sets, can improve the understanding of psychosis liability and its interactions with early trauma exposure. Building on previous genome-wide PRS research, our findings indicate that pathway-level genetic signals – particularly those related to glutamatergic and serotonergic systems – may contribute meaningfully to both trauma exposure and psychosis risk in a large, deeply phenotyped, multi-site case-control cohort.

Consistent with prior evidence that genetic risk influences environmental exposure through rGE mechanisms, we found that three of four pPGSs were positively associated with cumulative childhood trauma [21]. The serotonin pathway was most consistently associated with trauma, underscoring its potential role in early affective sensitivity and stress reactivity. These findings echo evidence linking serotonergic function to both internalising symptoms and trauma responsivity, particularly during early development [50]. To address potential bias due to pathway size (i.e., the number of genes contributing to each score), we additionally residualised the variance explained. The same serotonergic and glutamatergic pathways continued to show relatively greater enrichment after this adjustment, indicating that their associations were not simply attributable to pathway size.

When examining gene-environment interplay, we found evidence for significant interactions between both the dopamine and serotonin pPGSs and trauma exposure in predicting psychosis risk. Specifically, these negative interaction coefficients indicate that the strength of the association between trauma and psychosis decreased as polygenic liability increased. Although these effects were small in magnitude, they represent changes of roughly 10-120% in predicted odds of psychosis across the observed range of trauma exposure, consistent with typical GxE effect sizes in complex traits. In other words, at higher levels of genetic risk, additional trauma exposure contributed proportionally less to psychosis likelihood [25, 51]. Importantly, such attenuation patterns should be interpreted with caution. Negative interaction terms in logistic regression models may arise from various statistical artifacts, including ceiling effects, scale dependence, non-linearity, or residual confounding, rather than reflecting true biological saturation effects. These findings do not provide direct mechanistic evidence but rather suggest that the relative contribution of early adversity to psychosis risk varies across levels of genetic liability [25, 51]. This interpretation is consistent with previous observations that schizophrenia PRS exhibited stronger effects among individuals without severe environmental risk exposures, such as childhood adversity or frequent cannabis use [52].

While comparable interaction patterns emerged across trauma indices, the strongest and most consistent effects were observed for the dopaminergic and serotonergic pathways, both showing significant negative interactions with composite and neglect-only scores. The GABA pathway showed only a weak trend toward interaction with abuse-related trauma, which did not survive FDR correction, and no significant differences were detected between abuse and neglect exposures in pairwise comparisons. Thus, while there is some indication that GABAergic mechanisms might be more relevant to trauma types involving active threat or harm, this interpretation remains speculative. The glutamatergic pathway, despite contributing substantially to genetic variance in psychosis and trauma exposure, showed no consistent evidence of moderation effects. Together, these results indicate that dopaminergic, serotonergic, and to a lesser extent GABAergic pathways may moderate the impact of early traumatic experiences on psychosis risk, whereas glutamatergic variation showed no consistent evidence of interaction.

Importantly, these associations and interactions remained after adjusting for the genome-wide psychosis PRS, suggesting that they are not merely driven by overall polygenic liability but reflect pathway-specific effects within neurotransmitter systems. Consistent patterns were observed when using raw continuous CTQ scores in sensitivity analysis, supporting the robustness of the findings across trauma operationalisations.

An important consideration in interpreting the observed GxE effects is the presence of rGE, particularly for the dopaminergic and serotonergic pPGSs, which were associated with childhood trauma exposure. When genetic liability influences environmental exposure, interaction effects may partly reflect overlapping pathways of risk rather than true moderation. Notably, the glutamatergic pPGS showed robust rGE with trauma but no evidence of GxE, suggesting dissociable mechanisms. The issue was further examined in sensitivity analyses in which trauma scores were residualised on the corresponding pPGS prior to testing GxE, with similar results reported in the Supplementary Material.

Recent studies have begun to demonstrate the value of pPGSs for investigating psychosis risk mechanisms. Tubbs et al. [53] showed that pPGSs can capture biologically interpretable variance beyond genome-wide scores [53], while Warren et al. [6] linked neurotransmitter-specific pPGSs to distinct symptom and cognitive profiles in early psychosis [6]. Consistent with these findings, our results support the hypothesis that serotonergic and dopaminergic genetic liability contributes to psychosis susceptibility through partially distinct mechanisms, here interacting with early environmental stressors. Unlike prior studies, we extend the application of pPGSs to gene-environment correlation and interaction.

These novel findings have several implications. First, they provide proof-of-concept that polygenic scores defined by neurotransmitter pathway genes and derived from GWAS reflecting a broader psychosis phenotype than just schizophrenia, may capture meaningful psychiatric risk beyond conventional genome-wide scores. Second, they support the hypothesis that biological systems implicated in psychosis may also shape vulnerability to early adversity. Third, they demonstrate that integrating pPGSs with trauma exposure reveals meaningful gene-environment interactions, highlighting that genetic liability and early adversity jointly, rather than independently, shape psychosis risk [54].

Nonetheless, limitations should be acknowledged. Pathway curation relied on publicly available databases, which may not capture dynamic gene regulation or functional variation, pPGSs were constructed using transdiagnostic GWAS summary statistics, which can conceal disorder-specific signals. Trauma exposure was retrospectively assessed, which introduces potential recall bias, although similar trends have been observed in prospective cohorts [55, 56]. Effect sizes were modest throughout, as expected for polygenic influences and complex interactions, underscoring that these findings are mechanistically informative rather than predictive. Additionally, the available sample size limited the power of the analyses. External replication in larger cohorts is needed to evaluate generalisability and robustness of our findings. Future studies should extend pPGSs analyses to ancestrally diverse populations to improve equity in risk prediction. Finally, while the GxE findings are promising, further work is required to test causal mechanisms using longitudinal, mediation, and functional design.

In conclusion, these novel findings underscore the potential of pPGSs to clarify the biological mechanisms linking genetic liability and early environmental exposures in psychosis [57]. The observed dopaminergic and serotoninergic GxE effects suggest that trauma does not uniformly amplify genetic risk, but interacts with it in complex, possibly threshold-dependent ways. Importantly, these effects persisted after adjustment for genome-wide psychosis PRS, and were consistent across alternative trauma measures, reinforcing robustness. Incorporating DNA methylation and other epigenetic biomarkers could help define the pathways through which genetic predisposition and environmental adversity jointly shape developmental trajectories and psychiatric vulnerability. Ultimately, combining polygenic, epigenetic, and environmental data may pave the way for multilevel risk models with improved specificity, mechanistic insight, and translational value.

## Supplementary information

Supplementary Material

Pathway membership for each gene.

Genes assigned to each signaling pathway (dopamine, serotonin, GABA, glutamate).

## Data Availability

De-identified data may be made available upon reasonable request, subject to approval by the relevant data access and ethics committees and in accordance with the EU-GEI data-sharing policies.

## References

[1] Craddock N, O’Donovan MC, Owen MJ. Psychosis genetics: modeling the relationship between schizophrenia, bipolar disorder, and mixed (or “Schizoaffective”) psychoses. Schizophr Bull. 2009;35:482–90. 19329560 10.1093/schbul/sbp020 PMC2669589

[2] Murray GK, Lin T, Austin J, McGrath JJ, Hickie IB, Wray NR. Could polygenic risk scores be useful in psychiatry?: A review. JAMA Psychiatry. 2021;78:210–9. 33052393 10.1001/jamapsychiatry.2020.3042

[3] Bogdan R, Baranger DAA, Agrawal A. Polygenic risk scores in clinical psychology: bridging genomic risk to individual differences. Annu Rev Clin Psychol. 2018;14:119–57. 29579395 10.1146/annurev-clinpsy-050817-084847 PMC7772939

[4] Choi SW, Garcia-Gonzalez J, Ruan Y, Wu HM, Johnson J, Hoggart C et al. The power of pathway-based polygenic risk scores. 2021. 10.21203/RS.3.RS-643696/V1. 10.1371/journal.pgen.1010624 PMC9937466 36749789

[5] Choi SW, García-González J, Ruan Y, Wu HM, Porras C, Johnson J, et al. PRSet: Pathway-based polygenic risk score analyses and software. PLoS Genet. 2023;19:e1010624. 36749789 10.1371/journal.pgen.1010624 PMC9937466

[6] Warren TL, Tubbs JD, Lesh TA, Corona MB, Pakzad SS, Albuquerque MD, et al. Association of neurotransmitter pathway polygenic risk with specific symptom profiles in psychosis. Mol Psychiatry. 2024;29:2389–98. 10.1038/s41380-024-02457-0. 38491343 10.1038/s41380-024-02457-0 PMC11412910

[7] Panov G, Panova P. Neurobiochemical disturbances in psychosis and their implications for therapeutic Intervention. Curr Top Med Chem. 2024;24:1784–98. 38265370 10.2174/0115680266282773240116073618

[8] Amato D, Canneva F, Cumming P, Maschauer S, Groos D, Dahlmanns JK, et al. A dopaminergic mechanism of antipsychotic drug efficacy, failure, and failure reversal: the role of the dopamine transporter. Mol Psychiatry. 2018;25:2101–18. 30038229 10.1038/s41380-018-0114-5 PMC7473845

[9] Kantrowitz JT, Javitt DC. N-methyl-d-aspartate (NMDA) receptor dysfunction or dysregulation: the final common pathway on the road to schizophrenia?. Brain Res Bull. 2010;83:108–21. 20417696 10.1016/j.brainresbull.2010.04.006 PMC2941541

[10] Coyle JT. The glutamatergic dysfunction hypothesis for schizophrenia. Harv Rev Psychiatry. 1996;3:241–53. 9384954 10.3109/10673229609017192

[11] Tang X, Jaenisch R, Sur M. The role of GABAergic signalling in neurodevelopmental disorders. Nat Rev Neurosci 2021 22:5. 2021;22:290–307. 10.1038/s41583-021-00443-x PMC9001156 33772226

[12] Inan M, Petros TJ, Anderson SA. Losing your inhibition: Linking cortical GABAergic interneurons to schizophrenia. Neurobiol Dis. 2013;53:36–48. 23201207 10.1016/j.nbd.2012.11.013 PMC3593807

[13] Di Giovanni G, Esposito E, Di Matteo V, Vincenzo Di Matteo C, di Ricerche Farmacologiche I, Negri M. Role of serotonin in central dopamine dysfunction. CNS Neurosci Ther. 2010;16:179–94. 20557570 10.1111/j.1755-5949.2010.00135.x PMC6493878

[14] Ciranna L. Serotonin as a modulator of glutamate- and GABA-mediated neurotransmission: implications in physiological functions and in pathology. Curr Neuropharmacol. 2006;4:101–14. 18615128 10.2174/157015906776359540 PMC2430669

[15] Scangos KW, State MW, Miller AH, Baker JT, Williams LM. New and emerging approaches to treat psychiatric disorders. Nat Med. 2023;29:317–33. 36797480 10.1038/s41591-022-02197-0 PMC11219030

[16] Alameda L, Christy A, Rodriguez V, Salazar De Pablo G, Thrush M, Shen Y, et al. Association between specific childhood adversities and symptom dimensions in people with psychosis: systematic review and meta-analysis. Schizophr Bull. 2021;47:975–85. 33836526 10.1093/schbul/sbaa199 PMC8266673

[17] Christy A, Cavero D, Navajeeva S, Murray-O’Shea R, Rodriguez V, Aas M, et al. Association between childhood adversity and functional outcomes in people with psychosis: a meta-analysis. Schizophr Bull. 2022;49:285–96. 10.1093/SCHBUL/SBAC105. 10.1093/schbul/sbac105 PMC10016406 36107860

[18] Zhou L, Sommer IEC, Yang P, Sikirin L, van Os J, Bentall RP, et al. What do four decades of research tell us about the association between childhood adversity and psychosis: an updated and extended multi-level meta-analysis. Am J Psychiatry. 2025;182:360–72. 40165558 10.1176/appi.ajp.20240456

[19] Flinn A, Hefferman-Clarke R, Parker S, Allsopp K, Zhou L, Begemann M, et al. Cumulative exposure to childhood adversity and risk of adult psychosis: a dose–response meta-analysis. Psychol Med. 2025;55:e162. 40438025 10.1017/S0033291725001138 PMC12150334

[20] Varese F, Smeets F, Drukker M, Lieverse R, Lataster T, Viechtbauer W, et al. Childhood adversities increase the risk of psychosis: a meta-analysis of patient-control, prospective-and cross-sectional cohort studies. Schizophr Bull. 2012;38:661–71. 22461484 10.1093/schbul/sbs050 PMC3406538

[21] Woolway GE, Smart SE, Lynham AJ, Lloyd JL, Owen MJ, Jones IR, et al. Schizophrenia polygenic risk and experiences of childhood adversity: a systematic review and meta-analysis. Schizophr Bull. 2022;48:967–80. 35674151 10.1093/schbul/sbac049 PMC9434424

[22] Warrier V, Kwong ASF, Luo M, Dalvie S, Croft J, Sallis HM, et al. Gene–environment correlations and causal effects of childhood maltreatment on physical and mental health: a genetically informed approach. Lancet Psychiatry. 2021;8:373–86. 33740410 10.1016/S2215-0366(20)30569-1 PMC8055541

[23] Trubetskoy V, Pardiñas AF, Qi T, Panagiotaropoulou G, Awasthi S, Bigdeli TB, et al. Mapping genomic loci implicates genes and synaptic biology in schizophrenia. Nature. 2022;604:502–8. 35396580 10.1038/s41586-022-04434-5 PMC9392466

[24] Mullins N, Forstner AJ, O’Connell KS, Coombes B, Coleman JRI, Qiao Z, et al. Genome-wide association study of more than 40,000 bipolar disorder cases provides new insights into the underlying biology. Nat Genet. 2021;53:817–29. 34002096 10.1038/s41588-021-00857-4 PMC8192451

[25] Alameda L, Rodriguez V, Di Forti M, Spinazzola E, Trotta G, Arango C, et al. The effect of polygenic risk score and childhood adversity on transdiagnostic symptom dimensions at first-episode psychosis: evidence for an affective pathway to psychosis. Transl Psychiatry. 2024;14:1–7. 39461938 10.1038/s41398-024-03149-7 PMC11513137

[26] Di Forti M, Quattrone D, Freeman TP, Tripoli G, Gayer-Anderson C, Quigley H, et al. The contribution of cannabis use to variation in the incidence of psychotic disorder across Europe (EU-GEI): a multicentre case-control study. Lancet Psychiatry. 2019;6:427–36. 30902669 10.1016/S2215-0366(19)30048-3 PMC7646282

[27] Gayer-Anderson C, Jongsma HE, Di Forti M, Quattrone D, Velthorst E, de Haan L, et al. The EUropean network of national schizophrenia networks studying gene-environment interactions (EU-GEI): incidence and first-episode case-control programme. Soc Psychiatry Psychiatr Epidemiol. 2020;55:645–57. 31974809 10.1007/s00127-020-01831-x

[28] Quattrone D, Di Forti M, Gayer-Anderson C, Ferraro L, Jongsma HE, Tripoli G, et al. Transdiagnostic dimensions of psychopathology at first episode psychosis: findings from the multinational EU-GEI study. Psychol Med. 2019;49:1378–91. 30282569 10.1017/S0033291718002131 PMC6518388

[29] Jongsma HE, Gayer-Anderson C, Lasalvia A, Quattrone D, Mulè A, Szöke A, et al. Treated incidence of psychotic disorders in the multinational EU-GEI study. JAMA Psychiatry. 2018;75:36–46. 29214289 10.1001/jamapsychiatry.2017.3554 PMC5833538

[30] McGuffin P, Farmer A, Harvey I. A polydiagnostic application of operational criteria in studies of psychotic illness: development and reliability of the OPCRIT system. Arch Gen Psychiatry. 1991;48:764–70. 1883262 10.1001/archpsyc.1991.01810320088015

[31] Bernstein DP, Fink L, Handelsman L, Foote J, Lovejoy M, Wenzel K, et al. Initial reliability and validity of a new retrospective measure of child abuse and neglect. Am J Psychiatry. 1994;151:1132–6. 8037246 10.1176/ajp.151.8.1132

[32] Perroud N, Paoloni-Giacobino A, Prada P, Olié E, Salzmann A, Nicastro R, et al. Increased methylation of glucocorticoid receptor gene (NR3C1) in adults with a history of childhood maltreatment: a link with the severity and type of trauma. Transl Psychiatry. 2011;1:e59–e59. 22832351 10.1038/tp.2011.60 PMC3309499

[33] Alameda L, Liu Z, Sham PC, Aas M, Trotta G, Rodriguez V, et al. Exploring the mediation of DNA methylation across the epigenome between childhood adversity and First Episode of Psychosis-findings from the EU-GEI study. Mol Psychiatry. 2023;28:2095–106. 37062770 10.1038/s41380-023-02044-9

[34] Huang MC, Schwandt ML, Ramchandani VA, George DT, Heilig M. Impact of multiple types of childhood trauma exposure on risk of psychiatric comorbidity among alcoholic inpatients. Alcohol Clin Exp Res. 2012;36:598. 10.1111/j.1530-0277.2011.01695.x PMC3370064 22420670

[35] Altshuler DL, Durbin RM, Abecasis GR, Bentley DR, Chakravarti A, Clark AG, et al. A map of human genome variation from population-scale sequencing. Nature. 2010;467:1061–73. 20981092 10.1038/nature09534 PMC3042601

[36] Ge T, Chen CY, Ni Y, Feng YCA, Smoller JW. Polygenic prediction via Bayesian regression and continuous shrinkage priors. Nat Commun. 2019;10:1–10. 30992449 10.1038/s41467-019-09718-5 PMC6467998

[37] Willer CJ, Li Y, Abecasis GR. METAL: fast and efficient meta-analysis of genomewide association scans. Bioinformatics. 2010;26:2190–1. 20616382 10.1093/bioinformatics/btq340 PMC2922887

[38] Bulik-Sullivan B, Loh PR, Finucane HK, Ripke S, Yang J, Patterson N, et al. LD Score regression distinguishes confounding from polygenicity in genome-wide association studies. Nat Genet. 2015;47:291–5. 25642630 10.1038/ng.3211 PMC4495769

[39] Van Bergen AH, Verkooijen S, Vreeker A, Abramovic L, Hillegers MH, Spijker AT, et al. The characteristics of psychotic features in bipolar disorder. Psychol Med. 2019;49:2036–48. 30303059 10.1017/S0033291718002854

[40] Aminoff SR, Onyeka IN, Ødegaard M, Simonsen C, Lagerberg TV, Andreassen OA, et al. Lifetime and point prevalence of psychotic symptoms in adults with bipolar disorders: a systematic review and meta-analysis. Psychol Med. 2022;52:2413–25. 36016504 10.1017/S003329172200201X PMC9647517

[41] Chakrabarti S, Singh N. Psychotic symptoms in bipolar disorder and their impact on the illness: a systematic review. World J Psychiatry. 2022;12:1204–32. 36186500 10.5498/wjp.v12.i9.1204 PMC9521535

[42] Smigielski L, Papiol S, Theodoridou A, Heekeren K, Gerstenberg M, Wotruba D, et al. Polygenic risk scores across the extended psychosis spectrum. Transl Psychiatry. 2021;11:600. 10.1038/S41398-021-01720-0. 34836939 10.1038/s41398-021-01720-0 PMC8626446

[43] Lewis CM, Vassos E. Prospects for using risk scores in polygenic medicine. Genome Med. 2017;9:1–3. 29132412 10.1186/s13073-017-0489-y PMC5683372

[44] Ogata H, Goto S, Sato K, Fujibuchi W, Bono H, Kanehisa M. KEGG: kyoto encyclopedia of genes and genomes. Nucleic Acids Res. 1999;27:29–34. 9847135 10.1093/nar/27.1.29 PMC148090

[45] Joshi-Tope G, Gillespie M, Vastrik I, D’Eustachio P, Schmidt E, de Bono B, et al. Reactome: a knowledgebase of biological pathways. Nucleic Acids Res. 2005;33:D428–D432. 15608231 10.1093/nar/gki072 PMC540026

[46] Carbon S, Ireland A, Mungall CJ, Shu S, Marshall B, Lewis S, et al. AmiGO: online access to ontology and annotation data. Bioinformatics. 2009;25:288–9. 19033274 10.1093/bioinformatics/btn615 PMC2639003

[47] Austin-Zimmerman I, Li Q, Johnson E, Trotta G, Spinazzola E, Coleman J, et al. Genetic pathways point to the biology underlying the association between cannabis use disorder and psychosis. Biol Psychiatry: Glob Open Sci. 2026;6:100711. 42181294 10.1016/j.bpsgos.2026.100711 PMC13196363

[48] Salvatore JE, Aliev F, Bucholz K, Agrawal A, Hesselbrock V, Hesselbrock M, et al. Polygenic risk for externalizing disorders: gene-by-development and gene-by-environment effects in adolescents and young adults. Clin Psychological Sci. 2015;3:189–201. 10.1177/2167702614534211 PMC4371857 25821660

[49] Benjamini Y, Hochberg Y. Controlling the false discovery rate: a practical and powerful approach to multiple testing. J R Stat Society: Ser B. 1995;57:289–300.

[50] Dayer A. Serotonin-related pathways and developmental plasticity: relevance for psychiatric disorders. Dialogues Clin Neurosci. 2014;16:29–41. 24733969 10.31887/DCNS.2014.16.1/adayer PMC3984889

[51] Sideli L, Murray RM, Schimmenti A, Corso M, La Barbera D, Trotta A, et al. Childhood adversity and psychosis: a systematic review of bio-psycho-social mediators and moderators. Psychol Med. 2020;50:1761–82. 32624020 10.1017/S0033291720002172

[52] Rodriguez V, Alameda L, Aas M, Gayer-Anderson C, Trotta G, Spinazzola E, et al. Polygenic and polyenvironment interplay in schizophrenia-spectrum disorder and affective psychosis; the EUGEI first episode study. Schizophr Bull. 2025;51:1254–65. 39658350 10.1093/schbul/sbae207 PMC12414556

[53] Tubbs JD, Leung PBM, Zhong Y, Zhan N, Hui TCK, Ho KKY et al. Pathway-specific polygenic scores improve cross-ancestry prediction of psychosis and clinical outcomes. MedRxiv [Preprint]. 2023. Available from: https://www.medrxiv.org/content/10.1101/2023.09.01.23294957v1.

[54] Andreassen OA, Hindley GFL, Frei O, Smeland OB. New insights from the last decade of research in psychiatric genetics: discoveries, challenges and clinical implications. World Psychiatry. 2023;22:4–24. 36640404 10.1002/wps.21034 PMC9840515

[55] Danese A, Widom CS. The subjective experience of childhood maltreatment in psychopathology. JAMA Psychiatry. 2021;78:1307–8. 34643689 10.1001/jamapsychiatry.2021.2874

[56] Newbury JB, Arseneault L, Moffitt TE, Caspi A, Danese A, Baldwin JR, et al. Measuring childhood maltreatment to predict early-adult psychopathology: comparison of prospective informant-reports and retrospective self-reports. J Psychiatr Res. 2018;96:57–64. 28965006 10.1016/j.jpsychires.2017.09.020 PMC5725307

[57] Alameda L, Trotta G, Quigley H, Rodriguez V, Gadelrab R, Dwir D, et al. Can epigenetics shine a light on the biological pathways underlying major mental disorders?. Psychol Med. 2022;52:1645–65. 35193719 10.1017/S0033291721005559 PMC9280283

