# Supplementary material for: Integrating biological pathway polygenic scores and trauma in psychosis: findings from the EU-GEI study
Source: Transl Psychiatry. 2026 Aug 5;16:412. doi: 10.1038/s41398-026-04317-7 (PMC13470413; doi:10.1038/s41398-026-04317-7)
Supplement: Supplementary file 1 [file 41398_2026_4317_MOESM1_ESM.docx]

**Supplementary Material**

[1. Additional Methods	4](#_Toc227747112)

[1.1. Population Stratification and Ancestry Determination (PCA)	4](#_Toc227747113)

[**Figure S1.** *PCA of the full EU-GEI sample projected onto the 1000 Genomes reference populations.*	4](#_Toc227747114)

[1.2. Cross-disorder Meta-analysis of Schizophrenia and Bipolar I	5](#_Toc227747115)

[**Figure S2.** *Mirrored Manhattan Plot of Schizophrenia and Bipolar I Disorder GWAS.*	6](#_Toc227747116)

[**Figure S3.** *QQ Plot for combined psychosis meta-analysis.*	6](#_Toc227747117)

[1.3. PRS-CS and PRSet parameters	7](#_Toc227747118)

[2. Biological Pathway PRS Distributions	8](#_Toc227747119)

[**Figure S4.** *Distribution of Selected pPGS Across the EU-GEI Sample.*	8](#_Toc227747120)

[3. Correlation among pPGSs	9](#_Toc227747121)

[**Figure S5.** *Correlation Heatmap of pPGSs.*	9](#_Toc227747122)

[4. Distribution of Childhood Trauma measures	10](#_Toc227747123)

[**Table S1.** Distribution of participants across trauma score categories.	10](#_Toc227747124)

[**Figure S7.** *Sample sizes across trauma score categories.*	12](#_Toc227747125)

[5. Additional Findings – Variance Explained, pPGS Distribution, and Interactions	13](#_Toc227747126)

[5.1. Additional pPGSs – Trauma Associations	13](#_Toc227747127)

[**Figure S8A.** *Forest Plot of pPGS – Trauma Associations – Abuse-Only Score.*	14](#_Toc227747128)

[**Figure S8B.** *Forest Plot of pPGS – Trauma Associations – Neglect-Only Score.*	15](#_Toc227747129)

[5.2. Variance in Childhood Trauma Explained by pPGS	16](#_Toc227747130)

[**Table S2.** pPGSs and Trauma Exposure Associations.	16](#_Toc227747131)

[**Figure S9.** *Variance in Childhood Trauma Explained by Pathway-Based Polygenic Scores.*	17](#_Toc227747132)

[5.3. Group Differences in Pathway pPGSs	18](#_Toc227747133)

[**Figure S10.** *Distribution of Pathway-Based Polygenic Scores (pPGSs) by Case-Control Status.*	19](#_Toc227747134)

[5.4. Interaction Effects Between pPGSs and Childhood Trauma	20](#_Toc227747135)

[**Figure S11A.** *pPGS x Abuse-Only Score Interaction on Psychosis Risk.*	20](#_Toc227747136)

[**Figure S11B.** *pPGS x Neglect-Only Score Interaction on Psychosis Risk.*	21](#_Toc227747137)

[**Table S3.** Interaction Effects Between pPGSs and Childhood Trauma on Psychosis Risk.	22](#_Toc227747138)

[5.5. Model Performance: Nagelkerke R^2^	22](#_Toc227747139)

[**Table S4.** Hierarchical model variance explained (Nagelkerke R^2^) for Trauma, Psychosis PRS, and pPGSs.	23](#_Toc227747140)

[**Figure S12.** *Incremental Variance Explained (ΔR^2^) by pPGS.*	24](#_Toc227747141)

[6. Psychosis Polygenic Risk and Adjusted Pathway Analyses	25](#_Toc227747142)

[6.1. Psychosis PRS Analyses (rGE and GxE)	25](#_Toc227747143)

[6.2. Pathway Analyses Adjusted for Psychosis PRS	25](#_Toc227747144)

[**Table S5.** Pathway-specific associations (rGE) and interaction effects (GxE) adjusted for psychosis PRS.	26](#_Toc227747145)

[**Figure S13.** *Comparison of Unadjusted and Psychosis PRS-Adjusted Analyses for the Abuse-Only Score.*	27](#_Toc227747146)

[**Figure S14.** *Comparison of Unadjusted and Psychosis PRS-Adjusted Analyses for the Neglect-Only Score.*	28](#_Toc227747147)

[7. Sensitivity Analyses Using Raw Continuous CTQ Scores	29](#_Toc227747148)

[7.1. Gene – Environment Correlation (rGE)	29](#_Toc227747149)

[**Table S6.** pPGSs and CTQ Scores Associations.	29](#_Toc227747150)

[**Figure S15A.** *Forest Plot of pPGS – Trauma Associations – CTQ Total Score.*	30](#_Toc227747151)

[**Figure S15B.** *Forest Plot of pPGS – Trauma Associations – CTQ Abuse Score.*	30](#_Toc227747152)

[**Figure S15C.** *Forest Plot of pPGS – Trauma Associations – CTQ Neglect Score.*	31](#_Toc227747153)

[7.2. Gene x Environment Interaction (GxE)	31](#_Toc227747154)

[**Table S7.** Interaction Effects Between pPGSs and Childhood Trauma on Psychosis Risk.	31](#_Toc227747155)

[**Figure S16A.** *pPGS x CTQ Total Score Interaction on Psychosis Risk.*	33](#_Toc227747156)

[**Figure S16B.** *pPGS x CTQ Abuse Score Interaction on Psychosis Risk.*	34](#_Toc227747157)

[**Figure S16C.** *pPGS x CTQ Neglect Score Interaction on Psychosis Risk.*	35](#_Toc227747158)

[8. Sex-stratified Sensitivity Analyses	36](#_Toc227747159)

[**Table S8.** Sex-stratified Sensitivity Analyses of rGE and GxE.	36](#_Toc227747160)

[9. Sensitivity Analyses Accounting for rGE	37](#_Toc227747161)

[**Table S9.** Sensitivity analyses of pathway-specific GxE using residualised trauma scores.	37](#_Toc227747162)

1. Additional Methods

## *1.1. Population Stratification and Ancestry Determination (PCA)*

Genetic ancestry was determined using a stepwise procedure previously implemented in EU-GEI WP2 analyses. Genotype data were merged with Phase 3 of the 1000 Genome Project reference panel, and ancestry principal components (PCs) were derived from overlapping SNPs. Individuals were assigned to major superpopulations using clustering informed by 1000 Genomes reference samples, and only those clustering with the European (EUR) group were retained. Fine-scale population structure was further assessed using iterative pruning and unsupervised clustering (IPCAPS), allowing identification of homogeneous European ancestry clusters without a priori assumptions. Cluster-specific quality control and PCA were performed, and individuals not classified as European were excluded prior to downstream analyses. All regression models were adjusted for the first ten ancestry PCs to control for residual population stratification. Visualisation of the ancestry inference procedure, including projection of the full EU-GEI sample onto the 1000 Genomes reference PC space, is provided in **Figure** **S1**.

### **Figure S1.** *PCA of the full EU-GEI sample projected onto the 1000 Genomes reference populations.*

Principal component plot (PC1 vs. PC2) of the full EU-GEI genetic sample merged with Phase 3 of the 1000 Genomes Project reference panel. Coloured points represent 1000 Genomes reference individuals from the major superpopulations (AFR, AMR, EAS, EUR, SAS), while EU-GEI participants are overlaid as open black circles. The initial multi-ancestry sample comprised N = 1,593 individuals. Individuals clustering with the European (EUR) reference population were retained for downstream analyses (N = 1,192), whereas individuals clustering with non-European reference populations were excluded prior to analysis (N = 401).

## *1.2. Cross-disorder Meta-analysis of Schizophrenia and Bipolar I*

To derive a genetic measure of shared psychosis liability, we conducted a cross-disorder meta-analysis of GWAS summary statistics for schizophrenia and bipolar I disorder. This approach is motivated by epidemiological and genetic evidence indicating substantial overlap in liability across psychotic disorders and has been used in prior cross-disorder work within the Psychiatric Genomics Consortium (PGC).

Summary statistics were obtained from the PGC schizophrenia GWAS (PGC3; PMID: 35396580) and the PGC bipolar I disorder GWAS (PGC BD I; PMID: 34002096). EU-GEI samples were excluded from both GWAS prior to meta-analysis to avoid overlap between the training and target datasets used for PRS construction.

A fixed-effects, inverse-variance-weighted meta-analysis was performed using METAL. Only SNPs passing standard quality control in both input GWAS were retained. The final meta-analysis included 1,175,488 SNPs and comprised a total effective sample size of 328,423 individuals.

To assess potential inflation due to population stratification or sample overlap between the schizophrenia and bipolar I GWAS (e.g., shared controls), we applied LD Score Regression (LDSC) to the meta-analysis summary statistics. The observed-scale SNP heritability was 0.10 (SE = 0.003). The LDSC intercept was 1.21 (SE = 0.013), with a ratio of 0.139, indicating that approximately 14% of the inflation in test statistics was attributable to confounding, with the majority explained by true polygenic signal. These values are consistent with large-scale psychiatric GWAS and cross-disorder analyses.

We conducted a sensitivity analysis excluding SNPs with evidence of heterogeneity between schizophrenia and bipolar I (Cochran’s Q p < 0.05). After exclusion of heterogeneous SNPs, SNP heritability and LDSC intercept estimates were minimally altered, supporting the robustness of the meta-analytic results.

### **Figure S2.** *Mirrored Manhattan Plot of Schizophrenia and Bipolar I Disorder GWAS.*

Figure from Austin-Zimmerman et al., in press *Biological Psychiatry: Global Open Science.*

### **Figure S3.** *QQ Plot for combined psychosis meta-analysis.*

Figure from Austin-Zimmerman et al., in press *Biological Psychiatry: Global Open Science.*

## *1.3. PRS-CS and PRSet parameters*

PRS-CS was implemented using default MCMC settings (1,000 iterations; 500 burn-in) and automatic estimation of the global shrinkage parameter (phi = auto), with the 1000 Genomes Phase 3 European reference panel. Summary statistics from the cross-disorder psychosis meta-analysis described above were used as the training dataset. Posterior SNP effect sizes were applied to targeted genotype data using PLINK to derive individual-level scores.

pPGSs were generated using PRSice-2 (v.2.3.5) via the PRSet framework, with predefined SNP list; no additional clumping or p-value thresholding was applied.

2. Biological Pathway PRS Distributions

To evaluate the distribution of the biological pathway polygenic scores (pPGSs), we visualised the standardised scores for the four neurotransmitter systems used in our analyses: serotonin, dopamine, glutamate, and GABA. As shown in Figure S4, all pPGSs showed approximately normal distributions after z-score standardisation across the full analytic sample. Each panel displays a histogram of score values overlaid with a smoother kernel density curve, and a dashed vertical line marking the mean. While some distribution showed minor deviations, all pPGSs were sufficiently symmetric to be used as continuous predictors in regression models without requiring further transformation.

### **Figure S4.** *Distribution of Selected pPGS Across the EU-GEI Sample.*

Density plots and histograms showing the distribution of pPGS (z-scores) for major neurotransmitter systems: serotonin, dopamine, glutamate, and GABA. Scores were standardised across the analytic sample. Dashed vertical lines represent the mean (zero) of the standardised distributions.

3. Correlation among pPGSs

Pairwise Pearson correlations among pPGSs were examined descriptively to characterise shared genetic signal across neurotransmitters pathways (Figure S6). Correlations were moderate in magnitude (all r < 0.60), reflecting partial overlap among biologically related gene sets.

### **Figure S5.** *Correlation Heatmap of pPGSs.*

Pearson correlation coefficients (r) among pathway-based polygenic scores (pPGSs) for four major neurotransmitter systems (i.e., serotonin, dopamine, glutamate, and GABA). Warmer colours (yellow to red) indicate negative correlations, while cooler shades (purple to blue) indicate positive correlations. Values within each cell represent the strength of pairwise correlations.

4. Distribution of Childhood Trauma measures

To support the validity and interpretability of gene-environment analyses, we examined the distributions of all childhood trauma measures used in the main analyses, stratified by psychosis case-control status.

Violin plots illustrating the distribution of the composite trauma score, abuse-only, neglect-only score, and the continuous CTQ score are shown in Figure S6. Trauma scores were continuous and positively skewed, with higher median values observed among cases compared with controls across all trauma operationalisations. Non-parametric Wilcoxon rank-sum tests were used to compare distributions between cases and controls (all p < 0.001).

We also examined the number of participants contributing to each trauma score category for the composite, abuse-only, and neglect-only indices. Stacked bar plots stratified by case-control status are shown in Figure S7, and detailed counts are reported in Table S1. These distributions indicate that all trauma score categories were represented by sufficient numbers of participants.

### **Table S1.** Distribution of participants across trauma score categories.

| Trauma measure | Score category | N total | N controls | N cases | N Brazil | % Brazil |
| --- | --- | --- | --- | --- | --- | --- |
| Composite trauma score (0–5) | 0 | 793 | 568 | 225 | 199 | 25.1 |
| Composite trauma score (0–5) | 1 | 204 | 109 | 95 | 44 | 21.6 |
| Composite trauma score (0–5) | 2 | 108 | 48 | 60 | 30 | 27.8 |
| Composite trauma score (0–5) | 3 | 48 | 14 | 34 | 11 | 22.9 |
| Composite trauma score (0–5) | 4 | 29 | 14 | 15 | 13 | 44.8 |
| Composite trauma score (0–5) | 5 | 11 | 2 | 9 | 3 | 27.3 |
| Abuse-only score (0–3) | 0 | 940 | 645 | 295 | 226 | 24.0 |
| Abuse-only score (0–3) | 1 | 179 | 81 | 98 | 46 | 25.7 |
| Abuse-only score (0–3) | 2 | 54 | 24 | 30 | 22 | 40.7 |
| Abuse-only score (0–3) | 3 | 20 | 5 | 15 | 6 | 30.0 |
| Neglect-only score (0–2) | 0 | 908 | 630 | 278 | 235 | 25.9 |
| Neglect-only score (0–2) | 1 | 182 | 81 | 101 | 34 | 18.7 |
| Neglect-only score (0–2) | 2 | 103 | 44 | 59 | 31 | 30.1 |

**Figure S6.** *Distribution of CTQ total scores in cases and controls.*

Violin plots showing the distributions of CTQ total scores stratified by psychosis case-control status. Violin widths reflect kernel density estimates; black points indicate medians.

### **Figure S7.** *Sample sizes across trauma score categories.*

Stacked bar plots showing the number of participants contributing to each trauma score category for the abuse-only, composite, and neglect-only trauma indices. Bars are stratified by psychosis case-control status.

5. Additional Findings – Variance Explained, pPGS Distribution, and Interactions

## *5.1. Additional pPGSs – Trauma Associations*

As a sensitivity analysis, we repeated the pPGS – trauma association models using the Abuse-Only and Neglect-Only scores. These analyses allowed us to examine whether observed associations were specific to particular trauma subtypes. For the Abuse-Only Score, serotonin (β = 0.068, SE = 0.018, p < 0.001), dopamine (β = 0.047, SE = 0.018, p = 0.009), and glutamate (β = 0.043, SE = 0.018, p = 0.017) pPGSs were significantly associated with greater abuse exposure (**Figure S8A**). For the Neglect-Only Score, serotonin (β = 0.071, SE = 0.018, p < 0.001) and dopamine (β = 0.056, SE = 0.018, p = 0.002) pPGSs showed significant associations, glutamate showed a nominal association, and GABA was not significant (**Figure S8B**). Among all the pPGSs, the serotonin pPGS demonstrated the largest and most consistent associations across all three trauma definitions.

### **Figure S8A.** *Forest Plot of pPGS – Trauma Associations – Abuse-Only Score.*

Beta Estimate and 95% confidence intervals (CIs) from linear regression models examining associations between pPGSs and trauma exposure (Abuse Only). All models are adjusted for age, gender, study site, and the first 10 genetic principal components. Points represent beta coefficients, with horizontal bars indicating the 95% CI.

### **Figure S8B.** *Forest Plot of pPGS – Trauma Associations – Neglect-Only Score.*

Beta Estimate and 95% confidence intervals (CIs) from linear regression models examining associations between pPGSs and trauma exposure (Neglect Only). All models are adjusted for age, gender, study site, and the first 10 genetic principal components. Points represent beta coefficients, with horizontal bars indicating the 95% CI.

## *5.2. Variance in Childhood Trauma Explained by pPGS*

To evaluate whether pPGSs capture variance in childhood trauma exposure, we conducted linear regression models using each neurotransmitter-specific pPGS (dopamine, GABA, glutamate, and serotonin) as a predictor. Three trauma outcomes were considered separately: the broader Composite Trauma Score, the Abuse-Only Score, and the Neglect-Only Score. All models included sex, age, study site, and the first 10 ancestry principal components as covariates. Full regression results are presented in **Table S2**. We report both the unadjusted R^2^ and adjusted R^2^ values to account for potential inflation due to covariate inclusion and model complexity.

### **Table S2.** pPGSs and Trauma Exposure Associations.

| pPGS | Outcome | β | SE | p-value | R^2^ | Adj R^2^ | FDR p-value |
| --- | --- | --- | --- | --- | --- | --- | --- |
| *Serotonin* | *Composite Score* | 0.1389 | 0.0303 | < 0.001 | 0.0449 | 0.0303 | < 0.001 |
| *Dopamine* |  | 0.1023 | 0.0301 | < 0.001 | 0.0372 | 0.0225 | 0.0014 |
| *Glutamate* |  | 0.0745 | 0.0308 | 0.0157 | 0.0326 | 0.0178 | 0.0209 |
| *GABA* |  | 0.0435 | 0.0352 | 0.2162 | 0.029 | 0.0141 | 0.2162 |
| *Serotonin* | *Abuse-Only Score* | 0.0682 | 0.0179 | < 0.001 | 0.0445 | 0.0298 | < 0.001 |
| *Dopamine* |  | 0.0465 | 0.0178 | 0.0092 | 0.0382 | 0.0235 | 0.0184 |
| *Glutamate* |  | 0.0431 | 0.0182 | 0.018 | 0.0373 | 0.0225 | 0.024 |
| *GABA* |  | 0.0121 | 0.0208 | 0.5597 | 0.0329 | 0.0181 | 0.5597 |
| *Serotonin* | *Neglect-Only Score* | 0.0708 | 0.0179 | < 0.001 | 0.0357 | 0.0209 | < 0.001 |
| *Dopamine* |  | 0.0558 | 0.0178 | 0.0017 | 0.031 | 0.0161 | 0.0035 |
| *Glutamate* |  | 0.0315 | 0.0182 | 0.0835 | 0.0253 | 0.0104 | 0.1114 |
| *GABA* |  | 0.0314 | 0.0207 | 0.1299 | 0.0248 | 0.0098 | 0.1299 |

As shown in **Figure S9**, all four pPGSs explained a small but non-negligible portion of variance in trauma exposure. The serotonin pPGS accounted for the highest proportion of variance across both trauma measures, followed by dopamine and glutamate scores. The GABA pPGS contributed the least, and particularly for the Abuse-Only index. Overall, variance explained was consistently higher for the Composite Trauma Score than for the Abuse-Only and Neglect-Only Scores likely reflecting the inclusion of multiple domains that may be better align with polygenic liability for stress sensitivity and affective dysregulation.

To account for differences in pathway size, defined by the number of genes included in each score, we also computed size-adjustment residual R^2^ values by regressing raw R^2^ on pathway genes count within each trauma outcome. These adjusted metrics, also displayed in **Figure S9**, revealed that the magnitude of explained variance was not simply driven by pathway size, with serotonergic and glutamatergic pPGSs continuing to show relatively higher enrichment compared with dopaminergic and GABAergic pathways for the Composite and Abuse-Only indices, while Neglect-Only index showed a slightly different pattern with more modest and less differentiated effects across pathways.

### **Figure S9.** *Variance in Childhood Trauma Explained by Pathway-Based Polygenic Scores.*

Bar plots showing the variance explained (adjusted R², and size-adjusted R^2^) in childhood trauma scores by individual pathway-based polygenic scores (pPGSs) for dopamine, GABA, glutamate, and serotonin. Results are presented separately for the Composite Score (left panel), the Abuse-Only Score (central panel) and the Neglect-Only Score (left panel). Each pPGS was tested in a linear model adjusting for sex, age, study site, and 10 genetic principal components. Green bars indicate adjusted R², and orange bars indicate the size-adjusted R^2^ (residualised on pathway genes count and re-centred around the mean raw R^2^), representing variance explained after accounting for pathway size.

## *5.3. Group Differences in Pathway pPGSs*

To explore whether pPGSs differentiate individuals with psychosis from unaffected controls, we compared the distribution of each neurotransmitter-related pPGS (dopamine, GABA, glutamate, and serotonin) by diagnostic group. Standardised scores were plotted using violin plots, which visualise the full distribution of each pPGS along with embedded boxplots indicating the median and interquartile range (IQR) within each group (**Figure S10**).

Visual inspection suggests higher average scores among cases compared to controls across all four pathways, with the most noticeable shift observed for the glutamate and serotonin pPGS. These trends are consistent with our regression findings showing dose-response increases in predicted psychosis probability across pPGS quintiles. While individual group differences were modest, the overall pattern supports the hypothesis that biologically informed polygenic signals – especially those related to glutamatergic and serotonergic pathways – are elevated among individuals with psychotic disorders relative to controls.

These group-level trends further justify the inclusion of pPGSs as continuous predictors in downstream association and interaction models.

### **Figure S10.** *Distribution of Pathway-Based Polygenic Scores (pPGSs) by Case-Control Status.*

Violin plots illustrating the distributions of standardised pPGSs for dopamine, GABA, glutamate, and serotonin across case and control groups. Each plot displays the full density of the pPGS distribution with an embedded boxplot indicating the median and interquartile range (IQR).

## *5.4. Interaction Effects Between pPGSs and Childhood Trauma*

As a sensitivity analysis, we examined whether pPGS x trauma interactions differed when using Abuse-Only or Neglect-Only scores instead of the Composite measure. For the Abuse-Only Score, a nominal negative interactions was found for GABA pPGSs (**Figure S11A**).

### **Figure S11A.** *pPGS x Abuse-Only Score Interaction on Psychosis Risk.*

Predicted probability of psychosis across the full distribution of standardised biological pathway PRSs, stratified by level of trauma exposure. Each line represents a different trauma level (0-3 adversities). 95% confidence intervals are shaded. Higher abuse exposure (warmer colours) is associated with greater psychosis risk across all pathways. For the dopamine, serotonin, and GABA pathways, the slope of the association between pPGS and psychosis risk flattens at higher trauma levels, suggesting a negative GxE.

For the Neglect-Only Score, dopamine and serotonin pPGSs again showed robust negative interactions, whereas glutamate and GABA were not significant after multiple testing correction (**Figure S11B**).

### **Figure S11B.** *pPGS x Neglect-Only Score Interaction on Psychosis Risk.*

Predicted probability of psychosis across the full distribution of standardised biological pathway PRSs, stratified by level of trauma exposure. Each line represents a different trauma level (0-2 adversities). 95% confidence intervals are shaded. Across all pathways, higher trauma exposure is associate with greater psychosis risk (from blue to red lines). For the dopamine and serotonin pathways, individuals with greater genetic liability (higher pPGS values) show a flatter increase in psychosis probability at higher neglect levels, suggesting a negative GxE.

Across all trauma definitions, dopamine and serotonin pPGSs consistently moderated the trauma-psychosis association, with higher pathway-specific genetic liability attenuating the observed risk relationship.

The main GxE findings are presented and discussed in the manuscript. For transparency and completeness, full interaction model results are presented in **Table S3**.

### **Table S3.** Interaction Effects Between pPGSs and Childhood Trauma on Psychosis Risk.

| Term | β (SE) | OR [95% CI] | p | FDR p |
| --- | --- | --- | --- | --- |
| *Composite Score* | | | | |
| *Serotonin pPGS* | -0.137 (0.064) | 0.872 [0.769–0.988] | **0.031** | **0.042** |
| *Dopamine pPGS* | -0.204 (0.066) | 0.815 [0.717–0.927] | **0.002** | **0.003** |
| *Glutamate pPGS* | -0.056 (0.071) | 0.946 [0.823–1.087] | 0.434 | 0.434 |
| *Abuse-Only Score* | | | | |
| *Serotonin pPGS* | -0.131 (0.112) | 0.877 [0.704–1.093] | 0.242 | 0.277 |
| *Dopamine pPGS* | -0.200 (0.118) | 0.819 [0.650–1.031] | 0.089 | 0.118 |
| *Glutamate pPGS* | -0.069 (0.122) | 0.934 [0.735–1.186] | 0.574 | 0.574 |
| *GABA pPGS* | -0.258 (0.127) | 0.773 [0.603–0.990] | **0.042** | 0.067 |
| *Neglect-Only Score* | | | | |
| *Serotonin pPGS* | -0.232 (0.102) | 0.793 [0.650–0.969] | **0.023** | **0.031** |
| *Dopamine pPGS* | -0.328 (0.101) | 0.720 [0.591–0.878] | **0.001** | **0.002** |
| *Glutamate pPGS* | -0.074 (0.112) | 0.929 [0.746–1.156] | 0.508 | 0.564 |
| *GABA pPGS* | 0.071 (0.122) | 1.073 [0.844–1.364] | 0.564 | 0.564 |

## *5.5. Model Performance: Nagelkerke R^2^*

To evaluate the incremental predictive value of pPGSs in explaining psychosis case-control status, we examined model performance across a series of hierarchical logistic regressions. For each trauma exposure index (i.e., Composite, Abuse-Only, and Neglect-Only), we compared four nested models: (0) covariates only (i.e., age, sex, ten ancestry PCs); (1) covariates + trauma; (2) covariates + trauma + Psychosis PRS; and (3) covariates + trauma + Psychosis PRS + pPGS. Model improvement was assessed via Nagelkerke R^2^ (ΔR^2^) and likelihood ratio tests comparing Model 2 vs. Model 3. To account for potential confounding due to pathway size (i.e., number of genes included in each pathway), ΔR^2^ values were also adjusted for pathway size (ΔR^2^ residual).

As shown in **Table S4** and **Figure S12**, inclusion of the Psychosis PRS substantially improved model fit relative to trauma-only models. The addition of pPGSs led to further incremental increases in variance explained, particularly for the glutamate and serotonin pathways. These effects remained evident after adjustment for pathway size. Comparable trends were observed across all trauma indices, with glutamate consistently showing the largest ΔR^2^ and the most robust likelihood-ratio test significance (p < 0.001).

### **Table S4.** Hierarchical model variance explained (Nagelkerke R^2^) for Trauma, Psychosis PRS, and pPGSs.

| Trauma variable | Pathway | M0: Covariates only | M1: M0 + Trauma | M2: M1 + Psychosis PRS | M3: M2 + pPGS | ΔR² (M1 → M2) | ΔR² (M2 → M3) | LRT p (Pathway) |
| --- | --- | --- | --- | --- | --- | --- | --- | --- |
| *Composite Score* | Dopamine | 0.162 | 0.245 | 0.414 | 0.415 | 0.169 | 0.0 | 0.473 |
|  | GABA | 0.162 | 0.245 | 0.414 | 0.415 | 0.169 | 0.0 | 0.463 |
|  | Glutamate | 0.162 | 0.245 | 0.414 | 0.425 | 0.169 | 0.01 | < 0.001 |
|  | Serotonin | 0.162 | 0.245 | 0.404 | 0.415 | 0.169 | 0.001 | 0.228 |
| *Abuse-Only Score* | Dopamine | 0.162 | 0.225 | 0.404 | 0.405 | 0.179 | 0.0 | 0.378 |
|  | GABA | 0.162 | 0.225 | 0.404 | 0.405 | 0.179 | 0.0 | 0.415 |
|  | Glutamate | 0.162 | 0.225 | 0.404 | 0.415 | 0.179 | 0.01 | < 0.001 |
|  | Serotonin | 0.162 | 0.225 | 0.404 | 0.406 | 0.179 | 0.001 | 0.149 |
| *Neglect-Only Score* | Dopamine | 0.162 | 0.218 | 0.399 | 0.400 | 0.179 | 0.0 | 0.438 |
|  | GABA | 0.162 | 0.218 | 0.399 | 0.399 | 0.181 | 0.0 | 0.6 |
|  | Glutamate | 0.162 | 0.218 | 0.399 | 0.411 | 0.181 | 0.011 | < 0.001 |
|  | Serotonin | 0.162 | 0.218 | 0.399 | 0.401 | 0.181 | 0.002 | 0.155 |

Model 0 = Covariates only (i.e., age, sex, site, ten ancestry PCs); Model 1 = Model 0 + Trauma; Model 2 = Model 1 + Psychosis PRS; Model 3 = Model 2 + PRS + pPGS. ΔR² columns indicate the incremental variance explained when adding the Psychosis PRS (Model 1 → Model 2) and pPGS (Model 2 → Model 3). LRT *p* (Pathway) = *p-*value from the likelihood-ratio test comparing Model 2 vs. Model 3.

### **Figure S12.** *Incremental Variance Explained (ΔR^2^) by pPGS.*

Incremental variance explained by pPGS across trauma indices. Bars show additional variance in psychosis risk explained by each neurotransmitter pPGS after accounting for trauma exposure and Psychosis PRS. Green bars indicate the unadjusted ΔR^2^, while the orange bars show the size-adjusted (residualised) ΔR^2^ controlling for pathway size (genes count).

6. Psychosis Polygenic Risk and Adjusted Pathway Analyses

## *6.1. Psychosis PRS Analyses (rGE and GxE)*

Analyses using the genome-wide psychosis PRS showed significant positive associations with all Trauma dimensions. For the Composite Score, higher psychosis PRS was associated with greater trauma exposure. Similar associations were found for the Abuse-Only (β = 0.169, SE = 0.0327, p < 0.001, FDR < 0.001) and Neglect-Only (β = 0.1623, SE = 0.0327, p < 0.001, FDR < 0.001) indices. No significant psychosis PRS x Trauma interaction effects on psychosis risk were detected after FDR correction (all p > 0.05). These findings suggest that the higher genome-wide genetic liability to psychosis is associated with greater exposure to childhood trauma, but the psychosis PRS does not significantly moderate the trauma-psychosis relationship.

## *6.2. Pathway Analyses Adjusted for Psychosis PRS*

To assess whether the pathway-specific findings were independent of genome-wide psychosis liability, all rGE and GxE models were re-run including the psychosis PRS as an additional covariate. Results for the three trauma indices are presented in Table S5.

### **Table S5.** Pathway-specific associations (rGE) and interaction effects (GxE) adjusted for psychosis PRS.

| Pathway | Model | Trauma Index | β (SE) | p | FDR p | Direction |
| --- | --- | --- | --- | --- | --- | --- |
| Serotonin | | | | | | |
|  | rGE | *Composite Score* | 0.112 (0.030) | **< 0.001** | **< 0.001** | ↑ |
|  | rGE | *Abuse-Only Score* | 0.054 (0.018) | **0.0026** | **0.0103** | ↑ |
|  | rGE | *Neglect-Only Score* | 0.058 (0.018) | **0.0014** | **0.0055** | ↑ |
|  | G×E | *Composite Score* | -0.150 (0.068) | **0.0273** | 0.0639 | ↓ |
|  | G×E | *Abuse-Only Score* | -0.113 (0.117) | 0.3355 | 0.3835 | ↓ |
|  | G×E | *Neglect-Only Score* | -0.277 (0.110) | **0.0119** | **0.0306** | ↓ |
| Dopamine | | | | | | |
|  | rGE | *Composite Score* | 0.058 (0.031) | 0.0612 | 0.1224 | ↑ |
|  | rGE | *Abuse-Only Score* | 0.024 (0.018) | 0.2013 | 0.4026 | ↑ |
|  | rGE | *Neglect-Only Score* | 0.035 (0.018) | 0.0597 | 0.1195 | ↑ |
|  | G×E | *Composite Score* | -0.178 (0.071) | **0.0125** | 0.0501 | ↓ |
|  | G×E | *Abuse-Only Score* | -0.168 (0.126) | 0.1808 | 0.2411 | ↓ |
|  | G×E | *Neglect-Only Score* | -0.287 (0.112) | **0.0105** | **0.0306** | ↓ |
| Glutamate | | | | | | |
|  | rGE | *Composite Score* | 0.012 (0.033) | 0.715 | 0.7174 | ↑ |
|  | rGE | *Abuse-Only Score* | 0.012 (0.019) | 0.5393 | 0.5393 | ↑ |
|  | rGE | *Neglect-Only Score* | 0.000 (0.019) | 0.998 | 0.998 | - |
|  | G×E | *Composite Score* | -0.096 (0.076) | 0.2094 | 0.2261 | ↓ |
|  | G×E | *Abuse-Only Score* | -0.088 (0.129) | 0.4977 | 0.4977 | ↓ |
|  | G×E | *Neglect-Only Score* | -0.159 (0.121) | 0.187 | 0.2494 | ↓ |
| GABA | | | | | | |
|  | rGE | *Composite Score* | -0.013 (0.036) | 0.7174 | 0.7174 | ↓ |
|  | rGE | *Abuse-Only Score* | -0.018 (0.021) | 0.412 | 0.5393 | ↓ |
|  | rGE | *Neglect-Only Score* | 0.004 (0.021) | 0.834 | 0.998 | ↑ |
|  | G×E | *Composite Score* | -0.099 (0.082) | 0.2261 | 0.2261 | ↓ |
|  | G×E | *Abuse-Only Score* | -0.276 (0.139) | **0.0471** | 0.1884 | ↓ |
|  | G×E | *Neglect-Only Score* | 0.004 (0.134) | 0.9748 | 0.9748 | ↑ |

Overall, adjustment for the psychosis PRS attenuated most associations, but serotonergic and dopaminergic pPGSs remained significantly associated with trauma exposure and showed negative interaction effects on psychosis risk. Results for the Composite Score are summarised in the manuscript, while corresponding plots for the Abuse-Only and Neglect-Only scores are shown below (Figures S13-S14).

### **Figure S13.** *Comparison of Unadjusted and Psychosis PRS-Adjusted Analyses for the Abuse-Only Score.*

Points indicate beta estimates from rGE (Panel A) and GxE (Panel B) models, with horizontal bars representing 95% CIs. Blue points and CIs represent estimates from models adjusted for age, sex, study site, and the first 10 genetic PCS (unadjusted for psychosis PRS). Orange dots and CIs represent models that additionally included psychosis PRS as covariate.

### **Figure S14.** *Comparison of Unadjusted and Psychosis PRS-Adjusted Analyses for the Neglect-Only Score.*

Points indicate beta estimates from rGE (Panel A) and GxE (Panel B) models, with horizontal bars representing 95% CIs. Blue points and CIs represent estimates from models adjusted for age, sex, study site, and the first 10 genetic PCS (unadjusted for psychosis PRS). Orange dots and CIs represent models that additionally included psychosis PRS as covariate.

7. Sensitivity Analyses Using Raw Continuous CTQ Scores

To evaluate whether the main findings were dependent on the operationalisation of childhood trauma, we repeated all primary analyses using the raw continuous CTQ scores (i.e., total, abuse, and neglect) rather than the cumulative indices based on binary domain cut-offs. This approach captures variability across the full CTQ range (25 - 125) and provides a more granular measure of trauma exposure.

## *7.1. Gene – Environment Correlation (rGE)*

Linear regression models were fitted with each pPGS as predictor and CTQ scores as outcome (i.e., total score, abuse-only score, neglect-only score), controlling for age, sex, study site, and the first ten ancestry PCS. Results were consistent with those obtained using the cumulative indices (**Table S6** and **Figures S15A-C**).

### **Table S6.** pPGSs and CTQ Scores Associations.

| pPGS | Outcome | β coefficient | SE | p-value | R^2^ | Adj R^2^ | FDR p-value |
| --- | --- | --- | --- | --- | --- | --- | --- |
| *Serotonin* | *CTQ Total Score* | 1.4304 | 0.3377 | < 0.001 | 0.0445 | 0.0298 | < 0.001 |
| *Dopamine* |  | 1.155 | 0.3358 | < 0.001 | 0.0396 | 0.0248 | < 0.001 |
| *Glutamate* |  | 0.8272 | 0.3432 | 0.0161 | 0.0347 | 0.0199 | 0.0214 |
| *GABA* |  | 0.7557 | 0.3917 | 0.054 | 0.033 | 0.0181 | 0.054 |
| *Serotonin* | *CTQ Abuse Score* | 0.6369 | 0.19 | < 0.001 | 0.0494 | 0.0348 | 0.0033 |
| *Dopamine* |  | 0.4689 | 0.1888 | 0.0131 | 0.0453 | 0.0307 | 0.0175 |
| *Glutamate* |  | 0.4793 | 0.1924 | 0.0129 | 0.0454 | 0.0307 | 0.0175 |
| *GABA* |  | 0.349 | 0.2198 | 0.1127 | 0.0424 | 0.0277 | 0.1127 |
| *Serotonin* | *CTQ Neglect Score* | 0.7936 | 0.1907 | < 0.001 | 0.0523 | 0.0378 | < 0.001 |
| *Dopamine* |  | 0.6861 | 0.1894 | < 0.001 | 0.049 | 0.0344 | < 0.001 |
| *Glutamate* |  | 0.3479 | 0.1939 | 0.073 | 0.041 | 0.0262 | 0.073 |
| *GABA* |  | 0.4067 | 0.2211 | 0.0661 | 0.0411 | 0.0264 | 0.073 |

The magnitude of the effect was slightly higher when raw CTQ scores were used, reflecting the broader scale and variance of the untransformed CTQ outcomes. The direction and ranking of effects were identical across trauma definitions, showing the strongest and most consistent associations for the serotonin pathway, followed by dopamine and glutamate. The proportion of variance explained was modestly lower than that observed in the cumulative models, which likely reflects increased measurement variability in the raw scores. Overall, these findings demonstrate that the rGE pattern is robust across alternative operationalisations of trauma exposure, with serotonergic, dopaminergic, and glutamatergic liabilities consistently predicting higher CTQ scores.

### **Figure S15A.** *Forest Plot of pPGS – Trauma Associations – CTQ Total Score.*

Beta Estimate and 95% confidence intervals (Cis) from linear regression models examining associations between pPGSs and trauma exposure (CTQ Total Score). All models are adjusted for age, gender, study site, and the first 10 genetic principal components. Points represent beta coefficients, with horizontal bars indicating the 95% CI.

### **Figure S15B.** *Forest Plot of pPGS – Trauma Associations – CTQ Abuse Score.*

Beta Estimate and 95% confidence intervals (Cis) from linear regression models examining associations between pPGSs and trauma exposure (CTQ Abuse Score). All models are adjusted for age, gender, study site, and the first 10 genetic principal components. Points represent beta coefficients, with horizontal bars indicating the 95% CI.

### **Figure S15C.** *Forest Plot of pPGS – Trauma Associations – CTQ Neglect Score.*

Beta Estimate and 95% confidence intervals (Cis) from linear regression models examining associations between pPGSs and trauma exposure (CTQ Neglect Score). All models are adjusted for age, gender, study site, and the first 10 genetic principal components. Points represent beta coefficients, with horizontal bars indicating the 95% CI.

## *7.2. Gene x Environment Interaction (GxE)*

We next examined GxE using the raw continuous CTQ scores (i.e., total, abuse, and neglect) to test whether findings were sensitive to trauma operationalisation. Results are summarised in **Table S7**.

### **Table S7.** Interaction Effects Between pPGSs and Childhood Trauma on Psychosis Risk.

| Term | β (SE) | OR [95% CI] | p | FDR p |
| --- | --- | --- | --- | --- |
| CTQ Total Score | | | | |
| *Serotonin pPGS* | -0.014 (0.006) | 0.986 [0.974–0.998] | **0.0217** | **0.0289** |
| *Dopamine pPGS* | -0.022 (0.006) | 0.978 [0.966–0.990] | **< 0.001** | **< 0.001** |
| *Glutamate pPGS* | -0.008 (0.007) | 0.992 [0.980–1.005] | 0.2485 | 0.2485 |
| *GABA pPGS* | -0.009 (0.007) | 0.991 [0.978–1.005] | 0.2118 | 0.242 |
| CTQ Abuse Score | | | | |
| *Serotonin pPGS* | -0.016 (0.011) | 0.984 [0.963–1.005] | 0.1392 | 0.1591 |
| *Dopamine pPGS* | -0.025 (0.012) | 0.976 [0.954–0.998] | **0.0318** | **0.0424** |
| *Glutamate pPGS* | -0.006 (0.012) | 0.994 [0.972–1.017] | 0.6027 | 0.6027 |
| *GABA pPGS* | -0.026 (0.012) | 0.974 [0.951–0.997] | **0.0299** | **0.0424** |
| CTQ Neglect Score | | | | |
| *Serotonin pPGS* | -0.023 (0.010) | 0.977 [0.957–0.997] | **0.0257** | **0.0343** |
| *Dopamine pPGS* | -0.038 (0.010) | 0.963 [0.943–0.982] | **< 0.001** | **< 0.001** |
| *Glutamate pPGS* | -0.016 (0.011) | 0.984 [0.963–1.005] | 0.1412 | 0.1614 |
| *GABA pPGS* | -0.000 (0.012) | 1.000 [0.977–1.023] | 0.9782 | 0.9782 |

Overall, the pattern of GxE effects was highly consistent with that observed in the main analyses based on composite scores. The serotonin and dopamine pPGSs again showed significant negative interactions with CTQ scores, indicating that higher neurotransmitter genetic liability attenuated the positive association between childhood trauma and psychosis risk. Directionality of the effects remained identical, with higher pPGS values flattening the trauma-psychosis slope (**Figures** **S16A-C**). Taken together, these sensitivity analyses demonstrate that the GxE are robust independently on the way childhood trauma is measured, reinforcing the reliability of the observed patterns.

### **Figure S16A.** *pPGS x CTQ Total Score Interaction on Psychosis Risk.*

Predicted probabilities of psychosis across the range of CTQ Total Scores, plotted separately for individuals at -1 SD (red), mean (green), and +1 SD (blue) levels of each pPGS. Shaded bands represent 95% CIs. From left to right, higher CTQ Total Scores indicate increasing exposure to childhood trauma. Across pathways, individuals with higher genetic liability (blue lines) generally show a flatter slope, suggesting a negative interaction between trauma and polygenic risk, most evident for the dopamine and serotonin pathways, where genetic loading appears to attenuate the trauma-psychosis association.

### **Figure S16B.** *pPGS x CTQ Abuse Score Interaction on Psychosis Risk.*

Predicted probabilities of psychosis across the range of CTQ Abuse Scores, plotted separately for individuals at -1 SD (red), mean (green), and +1 SD (blue) levels of each pPGS. Shaded bands represent 95% CIs. From left to right, higher CTQ Abuse Scores indicate increasing exposure to childhood trauma. Across pathways, individuals with higher genetic liability (blue lines) generally show a flatter slope, suggesting a negative interaction between trauma and polygenic risk, most evident for the dopamine, GABA and serotonin pathways, where genetic loading appears to attenuate the trauma-psychosis association.

### **Figure S16C.** *pPGS x CTQ Neglect Score Interaction on Psychosis Risk.*

Predicted probabilities of psychosis across the range of CTQ Neglect Scores, plotted separately for individuals at -1 SD (red), mean (green), and +1 SD (blue) levels of each pPGS. Shaded bands represent 95% CIs. From left to right, higher CTQ Neglect Scores indicate increasing exposure to childhood trauma. Across pathways, individuals with higher genetic liability (blue lines) generally show a flatter slope, suggesting a negative interaction between trauma and polygenic risk, most evident for the dopamine and serotonin pathways, where genetic loading appears to attenuate the trauma-psychosis association.

8. Sex-stratified Sensitivity Analyses

Sex-stratified sensitivity analyses were conducted for the pathway pPGSs showing significant rGE and GxE effects in the primary analyses (dopaminergic and serotonergic pathways – composite trauma score). Models were adjusted for age, study site, and the first 10 PCS. For rGE analyses, linear regression models were fitted with composite trauma score as outcome. For GxE analyses, logistic regression models predicted psychosis case-control status including main effects for trauma and pPGS and their interaction term. Results were broadly comparable across sex strata (**Table S8**). Although statistical power was reduced within groups (n = 568 females, and n = 624 males), no evidence was found that the observed associations were driven exclusively by one sex.

### **Table S8.** Sex-stratified Sensitivity Analyses of rGE and GxE.

| **Sex** | **Model** | **Trauma** | **pPGS** | **β** | **SE** | **p-value** |
| --- | --- | --- | --- | --- | --- | --- |
| *Female* | rGE | Composite Score | Dopamine | 0.1143 | 0.0481 | 0.0180 |
| *Male* |  |  | Dopamine | 0.0860 | 0.0384 | 0.0256 |
| *Female* |  |  | Serotonin | 0.1596 | 0.0468 | 0.0007 |
| *Male* |  |  | Serotonin | 0.1188 | 0.0398 | 0.0029 |
| *Female* | GxE |  | Dopamine | -0.2254 | 0.0915 | 0.0138 |
| *Male* |  |  | Dopamine | -0.1868 | 0.0980 | 0.0565 |
| *Female* |  |  | Serotonin | -0.2598 | 0.0859 | 0.0025 |
| *Male* |  |  | Serotonin | 0.0521 | 0.1104 | 0.6373 |

9. Sensitivity Analyses Accounting for rGE

Because several pPGSs, particularly dopaminergic and serotonergic, pathways, showed evidence of rGE with childhood trauma exposure, we conducted additional sensitivity analyses to evaluate whether the observed GxE effects were driven by rGE.

Specifically, childhood trauma scores were residualised on the corresponding pPGS and covariates prior to fitting interaction models. This procedure isolates the component of trauma not linearly predicted by genetic liability and allows assessment of whether GxE effects persist beyond rGE.

### **Table S9.** Sensitivity analyses of pathway-specific GxE using residualised trauma scores.

| Pathway | Trauma Index | β (SE) | p | FDR p |
| --- | --- | --- | --- | --- |
| Serotonin | *Composite Score* | -0.121 (0.065) | 0.0649 | 0.0865 |
|  | *Abuse-Only Score* | -0.104 (0.116) | 0.3718 | 0.4249 |
|  | *Neglect-Only Score* | -0.213 (0.104) | 0.0399 | 0.0532 |
| Dopamine | *Composite Score* | -0.208 (0.067) | 0.0019 | 0.0031 |
|  | *Abuse-Only Score* | -0.204 (0.119) | 0.0886 | 0.1181 |
|  | *Neglect-Only Score* | -0.330 (0.103) | 0.0013 | 0.0021 |
| Glutamate | *Composite Score* | -0.063 (0.071) | 0.3752 | 0.3753 |
|  | *Abuse-Only Score* | -0.091 (0.125) | 0.4630 | 0.4630 |
|  | *Neglect-Only Score* | 0.077 (0.112) | 0.4920 | 0.5623 |
| GABA | *Composite Score* | -0.076 (0.075) | 0.3098 | 0.3541 |
|  | *Abuse-Only Score* | -0.269 (0.129) | 0.0378 | 0.0604 |
|  | *Neglect-Only Score* | 0.054 (0.123) | 0.6571 | 0.6571 |

Residualised GxE analyses (**Table S9**) indicated that interaction effects involving dopaminergic pPGS and both composite and neglect-related trauma scores were similar in magnitude and direction relative to primary analyses. In contrast, serotonergic interaction effects were attenuated following residualisation, consistent with partial overlap between genetic influences on trauma exposure and moderation of psychosis risk. The glutamatergic pPGS showed evidence of rGE with trauma exposure but no GxE effects in either the primary or residualised analyses, suggesting pathway-specific mechanisms through which genetic liability contributes to psychosis risk.

Together, these findings indicate that the primary dopaminergic GxE effects are not explained by rGE.
